# Regenerative effects of platelet-rich plasma releasate injection in rabbit discs degenerated by intradiscal injection of condoliase

**DOI:** 10.1186/s13075-023-03200-w

**Published:** 2023-11-08

**Authors:** Takahiro Hasegawa, Koji Akeda, Junichi Yamada, Koki Kawaguchi, Norihiko Takegami, Tatsuhiko Fujiwara, Takahiro Natsume, Koichiro Ide, Yukihiro Matsuyama, Akihiro Sudo

**Affiliations:** 1 Department of Orthopaedic Surgery, Mie University Graduate School of Medicine, 2-174 Edobashi, Tsu City, Mie 514-8507 Japan; 2 Hamamatsu Pharma Research, Inc., Pharmacology, Hamamatsu, Shizuoka Japan; 3 Department of Orthopaedic Surgery, Hamamatsu University School of Medicine, Hamamatsu, Shizuoka Japan

**Keywords:** Intervertebral disc degeneration, Condoliase, Lumbar disc herniation, Platelet-rich plasma, Intradiscal injection therapy

## Abstract

**Background:**

Intradiscal condoliase injection is an alternative therapeutic option for lumbar disc herniation (LDH). However, it is often associated with disc degeneration. Several in vivo studies have demonstrated the regenerative potential of platelet-rich plasma (PRP) in disc degeneration. Thus, we hypothesized that the intradiscal injection of PRP releasate (PRPr), a soluble releasate isolated from PRP, has the potential to regenerate degenerated intervertebral discs (IVDs) induced by condoliase. This study examined the regenerative effects of PRPr on rabbit IVDs degenerated following condoliase injection.

**Methods:**

Eleven New Zealand white rabbits were used in this study. Condoliase (12.5 mU/10 μl) was injected into two non-contiguous discs (L2-L3 and L4-L5), and L3-L4 disc was left intact as a non-injection control. Saline (20 μl) or PRPr (20 μl) was randomly injected into L2-L3 and L4-L5 discs 4 weeks after the condoliase injection. Disc height (DH) was radiographically monitored biweekly from the day of condoliase injection to 16 weeks post-injection. Changes in DH were expressed as percentage DH (%DH) normalized to the baseline DH. Sixteen weeks after condoliase injection, all rabbits were euthanized, and subjected to MRI and histological analyses.

**Results:**

Intradiscal injection of condoliase induced a significant decrease in %DH (L2-L3 and L4-L5) to 52.0% at week 4. However, the %DH began to return to normal after saline injection and reached 76.3% at week 16. In the PRPr group, %DH began to recover to normal after the PRPr injection and was restored to 95.5% at week 16. The MRI-modified Pfirrmann grade of the PRPr group was significantly lower than that of the saline group (*P* < 0.01). Histological analyses showed progressive degenerative changes, including reduction of the NP area and condensation of the matrix in the saline and PRPr groups. The histological score of the PRPr group was significantly lower than that of the saline group (*P* < 0.01).

**Conclusions:**

PRPr has great potential to enhance the regeneration of degenerated rabbit IVDs induced by condoliase. The results of this preclinical study suggest that PRPr injection therapy may be indicated for patients with LDH who have poor recovery from disc degeneration after chemonucleolysis treatment with condoliase.

**Supplementary Information:**

The online version contains supplementary material available at 10.1186/s13075-023-03200-w.

## Background

The intervertebral disc (IVD), a fibrocartilaginous structure, is vital for the normal functioning of the spine. IVD degeneration is associated with several clinical conditions, including low back pain (LBP), disc herniation, spinal stenosis, and spinal deformities.

The IVD comprises a central gelatinous nucleus pulposus (NP) and the surrounding annulus fibrosus (AF). The NP comprises 85% of water and proteoglycans. The AF comprises an extracellular matrix (ECM) mixed with type 1 collagen fibers in the external layer and type 2 collagen fibers and chondrocytes in the inner layer. The mechanical function of the IVD depends strictly on the composition of its structure. NP can bear compressive loads owing to their intrinsic positive hydrostatic pressure, whereas AF can resist tensile stresses [1, 2].

Lumbar disc herniation (LDH) is one of the most common degenerative spinal diseases causing LBP and radiculopathy and significantly affects working-age patients [3, 4]. Acute lumbar radiculopathies due to herniated NP are primarily managed with conservative treatments, and many patients experience relief of symptoms within 6 to 12 weeks [3, 4]. However, surgery is recommended in cases that are resistant to conservative treatment. Surgical interventions such as discectomy provide faster pain relief and perceived recovery in patients with prolonged neurological symptoms. However, there are also risks of complications, such as postoperative cerebrospinal fluid leakage, nerve root injury, and postoperative pain [5].

Chemonucleolysis, an intermediate procedure between conservative and surgical treatments, has been clinically used to treat LDH [6]. Condoliase (Chondroitinase ABC) is an eliminase that degrades chondroitin sulfate and hyaluronic acid, which are glycosaminoglycans of proteoglycans abundant in the NP [7, 8]. Condoliase is a newly approved drug in Japan and a novel option for patients with insufficient symptom recovery after conservative treatment for LDH. The safety and significant improvement of neurological symptoms associated with LDH have recently been reported [9–11]. However, chemonucleolysis with condoliase has also been reported to be associated with decreased disc height, progressive disc degeneration, and LBP [11–13].

Platelet-rich plasma (PRP) contains autologous growth factors and cytokines and is widely used clinically for musculoskeletal disorders, including LBP due to disc degeneration [14]. It has been reported that the soluble releasate isolated from PRP (PRP releasate: PRPr) significantly stimulates cell proliferation and extracellular matrix metabolism in IVD cells in vitro [15]. After that, an intradiscal injection of PRPr was shown to restore structural changes in vivo using a rabbit annular puncture model [16]. A recent randomized controlled study showed that the intradiscal injection of PRPr was safe and improved LBP and LBP-related disabilities for 60 weeks [17]. Thus, we hypothesized that intradiscal injection of PRPr has the potential to regenerate the degenerated IVD induced by chemonucleolysis with condoliase.

This study aimed to determine the regenerative effects of the intradiscal administration of PRPr in rabbit IVDs degenerated following condoliase injection using radiological, magnetic resonance imaging (MRI), and histological analyses. The results of this preclinical study suggest that PRPr injection therapy may be indicated for patients with LDH who have poor recovery from disc degeneration after chemonucleolysis treatment with condoliase.

## Methods

### Animals

This study was conducted in strict accordance with the recommendations of the Guide for the Care and Use of Laboratory Animals of the National Institutes of Health. Eleven 16-week-old female New Zealand white rabbits (Japan SLC, Inc.), ranging from 2.7 to 3.2 kg in body weight, were used with the approval of our university’s Institutional Animal Care and Use Committee. Rabbits were housed in separate cages under standard conditions with a light–dark cycle (12 h-12 h) and dry-bulb room temperature at 22–24 °C and provided ad libitum access to tap water and food pellets daily.

### Preparation of allogenic PRP-releasate

Fresh blood (25 ml) was drawn from the inferior vena cava (IVC) of each rabbit using a 21-gauge needle into a syringe treated with 2.5 ml of anticoagulant (citrate dextrose-A solution; Termo, Tokyo, Japan). Allogeneic PRP was prepared using two centrifugation techniques, as previously reported [18]. Briefly, whole blood was centrifuged using a centrifugation apparatus (Himac CT 6D; Hitachi Ltd., Tokyo, Japan) for 15 min at 330 g. The plasma fraction was centrifuged for an additional 10 min at 1000 g. The supernatant plasma (platelet-poor plasma: PPP) was carefully removed, and the remaining PPP (approximately 1.0 mL) and precipitated platelets were designated as PRP. The number of platelets in whole blood and the PRP fraction was counted using a hemocytometer. To activate platelets, we treated PRP with 2% CaCl^2^ (Otsuka Pharmaceutical, Tokyo, Japan) for clot formation, followed by centrifugation at 1000 g for 10 min. The resulting soluble releasate from the clot preparation of PRP (PRPr) was isolated and kept at − 80 °C until use.

### Animal surgery

**Fig. 1:**
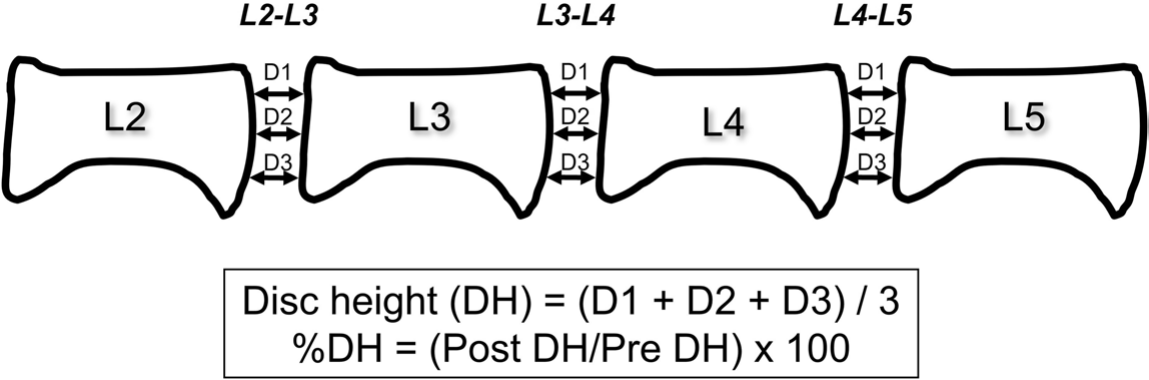
Radiographic measurement of disc height. The posterior disc height (D1), middle disc height (D2), and anterior disc height (D3) were measured and calibrated. The disc height (DH) was calculated as (D1 + D2 + D3)/3. Changes in DH were expressed as percentage DH (%DH) and normalized to the measured baseline DH as follows: %DH = (post-injection DH/baseline DH) × 100

Preoperatively, the rabbits were anesthetized by an intramuscular injection of ketamine hydrochloride (25 mg/kg; Ketalar®; Daiichi Sankyo, Tokyo, Japan) mixed with xylazine (5 mg/kg; Celactal®; Bayer, Tokyo, Japan). Lateral plain radiographs were obtained to determine baseline IVD height values before injection. Under general anesthesia with 3.0% isoflurane (Mylan Pharmaceutical Inc., Tampa, FL, USA), lumbar IVDs were exposed through a posterolateral approach; pharmaceutical-grade condoliase (Hernicore®; Daiichi Sankyo Company Limited, Tokyo, Japan) at a 12.5 mU/10 μl dose (same concentration as clinically used for patients with LDH) was injected using micro-syringes with a 30-gauge microneedle (outer diameter 0.52 mm, inner diameter 0.18 mm) (Ito Corporation, Fuji, Japan) into two non-contiguous discs (L2-L3 and L4-L5). The disc (L3-L4) between the injected discs was left intact as a control. Four weeks after the condoliase injection, saline (20 μl) or PRPr (20 μl) was randomly injected into L2-L3 and L4-L5 discs (Fig. 1). Condoliase (Hernicore®) was a gift from Daiichi Sankyo Company.

### Radiographic analysis of disc height

To assess the height of each disc, we obtained lateral radiographs of the lumbar segment immediately before surgery and at 2-week intervals until the animals were euthanized. A lateral lumbar soft radiograph was obtained under general anesthesia. A 50-mm metal wire was placed at the height of the rabbit spine in the lateral recumbent position as a calibration marker. All radiographic images were independently analyzed using image analysis software (ImageJ, US National Institutes of Health, Bethesda, MD, USA) by an orthopedic researcher who was blinded to the treatment groups. IVD height was measured as previously reported [19] with some modifications. The anterior disc height (D1), middle disc height (D2), and posterior disc height (D3) were measured and calibrated. The disc height (DH) was calculated as (D1 + D2 + D3)/3. Changes in DH were expressed as percentages of DH (%DH) and normalized to the measured baseline DH: %DH = (post-injection DH/baseline DH) × 100 (Fig. 1).

### MRI analysis

Sixteen weeks after the condoliase injection, all rabbits were euthanized, and the lumbar spinal columns and surrounding soft tissues were isolated and processed for MRI analysis. MRI was performed using a 3.0-Tesla imager (Ingenia 3.0 T; Philips, Amsterdam, The Netherlands) with a 3-inch birdcage extremity coil (Philips). The imaging protocol included sagittal T2W turbo-spine echo (TSE) with a repetition time (TR) of 3000 ms/echo and an echo time (TE) of 100 ms. The field of view (FOV) was 100 × 100 mm. All sagittal slice intervals were 3.0 mm thick, and five slices were available. T2-weighted images in the sagittal planes were obtained. The degree of disc degeneration was classified as grades 1–8 using the modified Pfirrmann grading system [20].

### Histological examination

Following MRI assessment, the experimental IVDs were excised from the vertebral body disc-vertebral body unit. Each IVD was fixed in 4% paraformaldehyde for 7 days at 4 °C and then permeated in an HCL-based decalcified reagent (K-CX, Falma, Inc., Tokyo, Japan) for 7 days. Midsagittal Sects. (5 μm) of each IVD were stained with hematoxylin, eosin, and Safranin O. An observer blinded to the experiment analyzed the histological sections and graded them using a recently established standardized histopathology scoring system for rabbit IVD degeneration [21]. Each histological sample was evaluated for the degeneration score in seven categories (I: NP shape, II: NP area, III: Matrix condensation, IV: Cell number, V: Border appearance, VI: AF morphology, and VII: EP) and for the repair score in two categories (VII: Cell cloning and IX: Cell morphology). The sum of the scores from I to VII was calculated as the degeneration score, and the sum from VII to IX was calculated as the repair score. The sum of degeneration and repair scores was calculated as the total score.

### Statistical analysis

Differences in DH and %DH were assessed for statistical significance using a two-way repeated-measures analysis of variance (ANOVA), followed by the Bonferroni post hoc test. The MRI-modified Pfirrmann grade and histological grading scores were assessed using the Kruskal–Wallis test for intergroup comparisons and the Friedman test for temporal changes. All data are expressed as mean ± standard deviation (SD). Correlations between the MRI-modified Pfirrmann grade and histological scores were assessed using Spearman’s rank correlation coefficient test. The accepted level of significance was *P* < 0.05. All statistical analyses were performed using the IBM Statistical Package for Social Sciences Software (SPSS) Statistics version 28.0 (IBM Japan, Tokyo).

## Results

### Platelet count

PRP platelet count was approximately 13.5 times higher than that of whole blood (whole blood: 233 × 103 /μl; PRP: 3157 × 103 /μl). No red blood cells or leukocytes were counted in the PRP.

### Change in DH

**Fig. 2:**
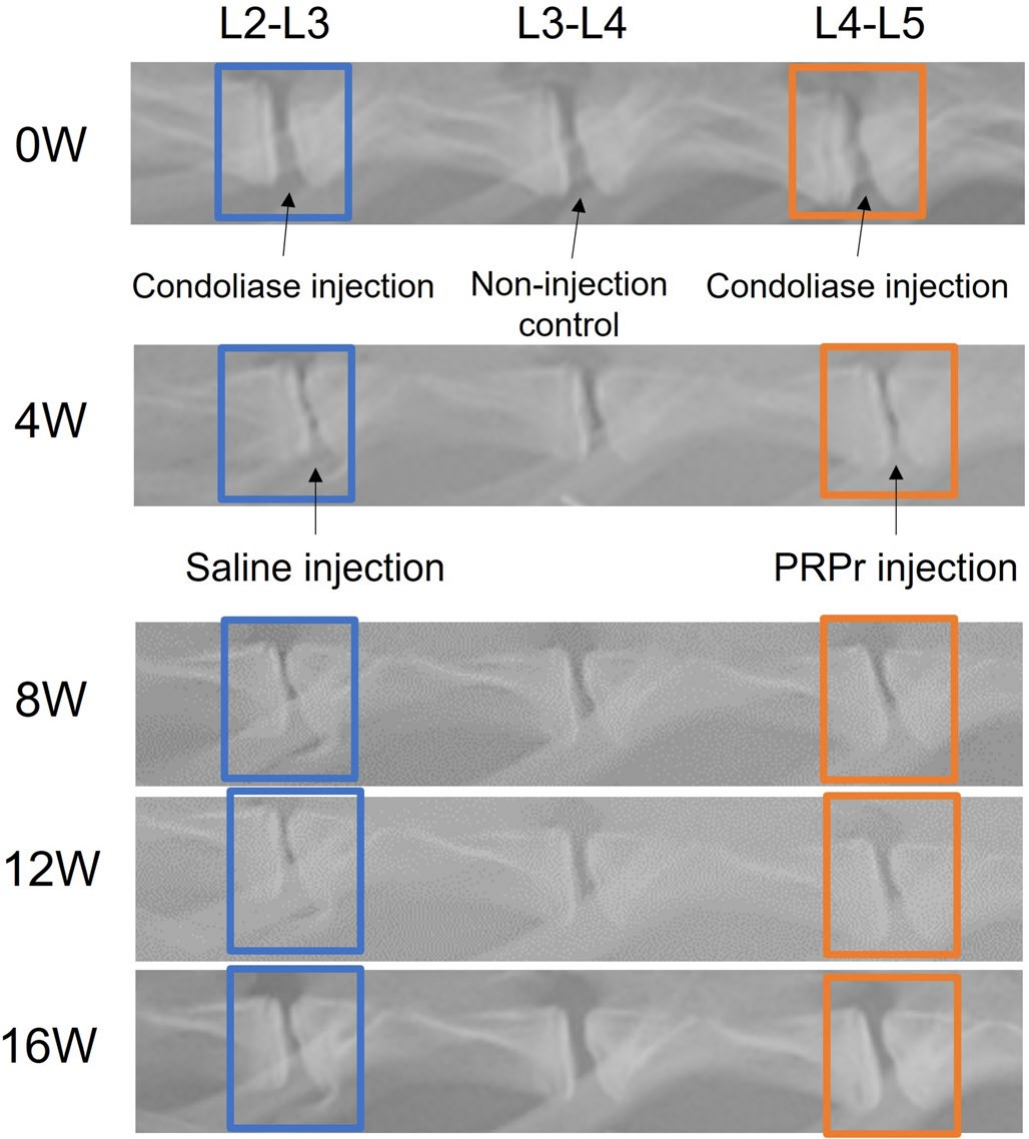
Lateral radiograms of a rabbit lumbar spine. Disc height (DH) was measured every 2 weeks using radiography in rabbit discs injected with condoliase. A significant decrease in DH on the lateral radiograph was observed 4 weeks after the condoliase injection. The radiogram of the PRPr group showed a more remarkable recovery of the disc space than that of the saline group at week 16

Radiographs taken 4 weeks after the injection of condoliase showed a significant narrowing of the DH compared to those of the pre-injection discs and intact non-punctured L3/L4 discs (Fig. 2). Radiographs at 8, 12, and 16 weeks showed a time-dependent recovery of DH after injection of both saline and PRPr; however, DH recovery was more prominent in the PRPr-injected disc than in the saline-injected disc (Fig. 2).

**Fig. 3:**
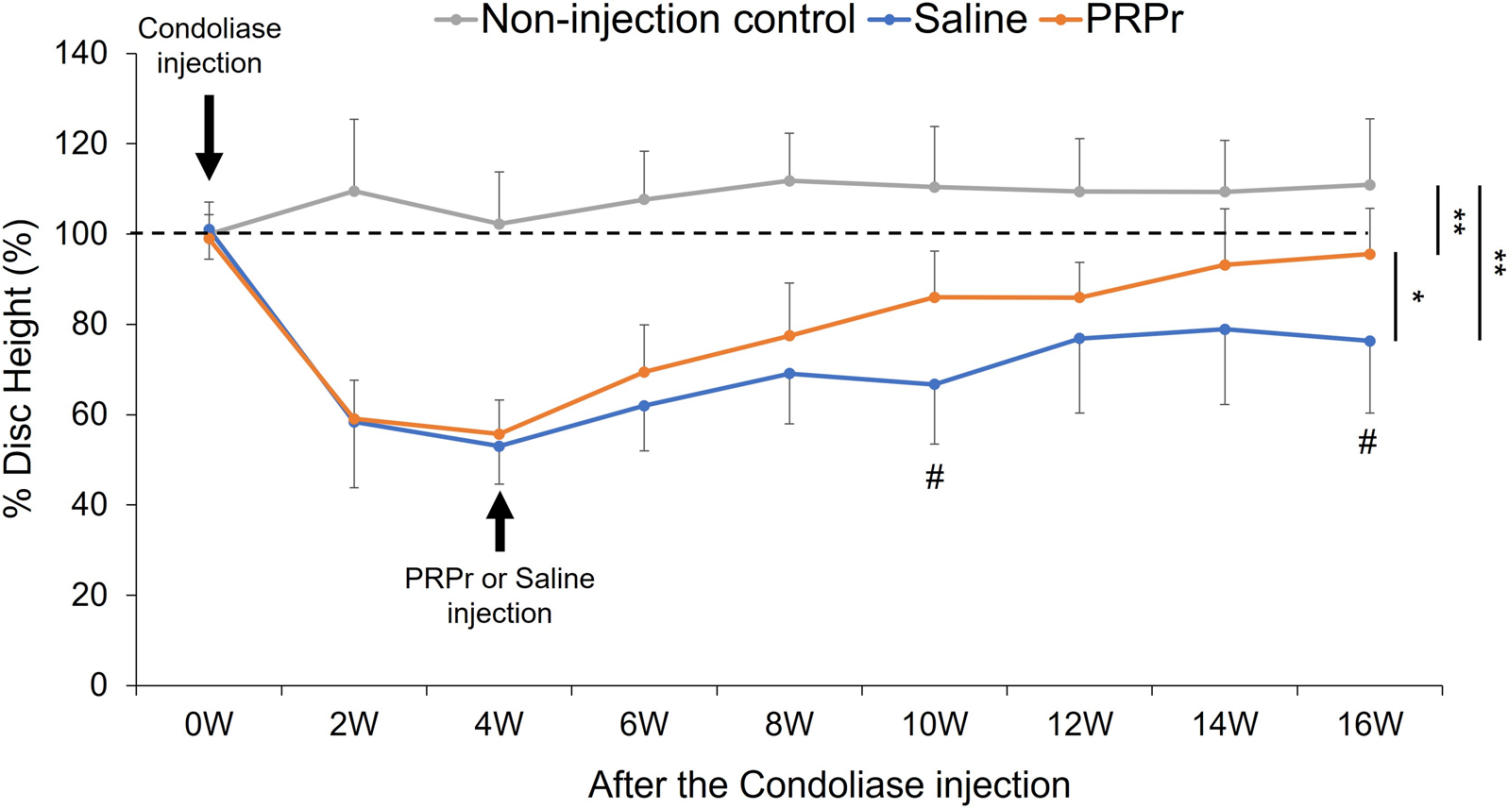
Change in percentage disc height (%DH). Intradiscal injection of condoliase induced a significant decrease in %DH at week 4. However, the injection of saline or PRPr resulted in a substantial recovery of %DH. Data are expressed as mean ± standard error of the mean (SEM). Two-way re-peated-measures ANOVA showed a significant difference among the non-injection control, saline, and PRPr groups (**P < 0.01, *P < 0.05). Time-point analysis showed that the %DH in the PRPr group was significantly higher than that in the saline group at weeks 10 and 16 (^#^*P* < 0.01)

Intradiscal injection of condoliase induced a significant decrease in %DH (L2-L3 and L4-L5) to 52.0 ± 8.1% at week 4 compared to that at baseline (*P* < 0.01; Fig. 3). However, the injection of saline or PRPr followed by condoliase injection resulted in significant recovery of the DH. The %DH began to recover after saline injection and reached 76.3 ± 16.0% at week 16. In the PRPr group, %DH started to recover to normal after PRPr injection and was restored to 95.5 ± 10.1% at week 16 (Fig. 3). Two-way repeated-measures ANOVA showed a significant difference between the two groups (*P* < 0.05). Time-point analysis showed that the %DH in the PRPr group was significantly higher than that in the saline group at week 10 (saline = 66.7 ± 13.2%, PRPr = 86.0 ± 10.2%, *P* < 0.01) and week 16 (saline = 76.3 ± 16.0%, PRPr = 95.5 ± 10.1%, *P* < 0.01) (Fig. 3).

### MRI grading

**Fig. 4:**
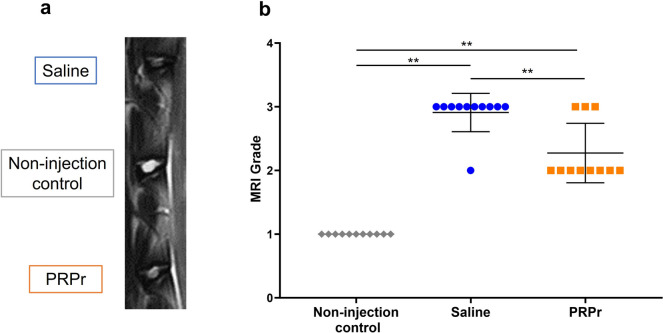
Magnetic resonance imaging (MRI) grading scores. MRI was performed 16 weeks after condoliase injection and 12 weeks after saline and platelet-rich plasma (PRP)-releasate injections. **a** Representative sagittal images of MRI. **b** MRI grading scores using the modified Pfirrmann grading system [20]. Data are presented as mean ± standard error of the mean (SEM). Non-injection control, *n* = 11 discs; saline, *n* = 11 discs; PRPr, *n* = 11 discs. **P < 0.01

MRI analysis was performed 16 weeks after the condoliase injections. Representative sagittal MRI images showed a higher intensity of T2-WI in the NP area in the discs injected with PRPr than in those injected with saline (Fig. 4a). The MRI grading scores of both the saline and PRPr groups were significantly higher than those of the control groups; however, the score of PRPr (2.2 ± 0.4) was significantly lower than that of the saline group (2.9 ± 0.3, *P* < 0.01) (Fig. 4b).

**Fig. 5:**
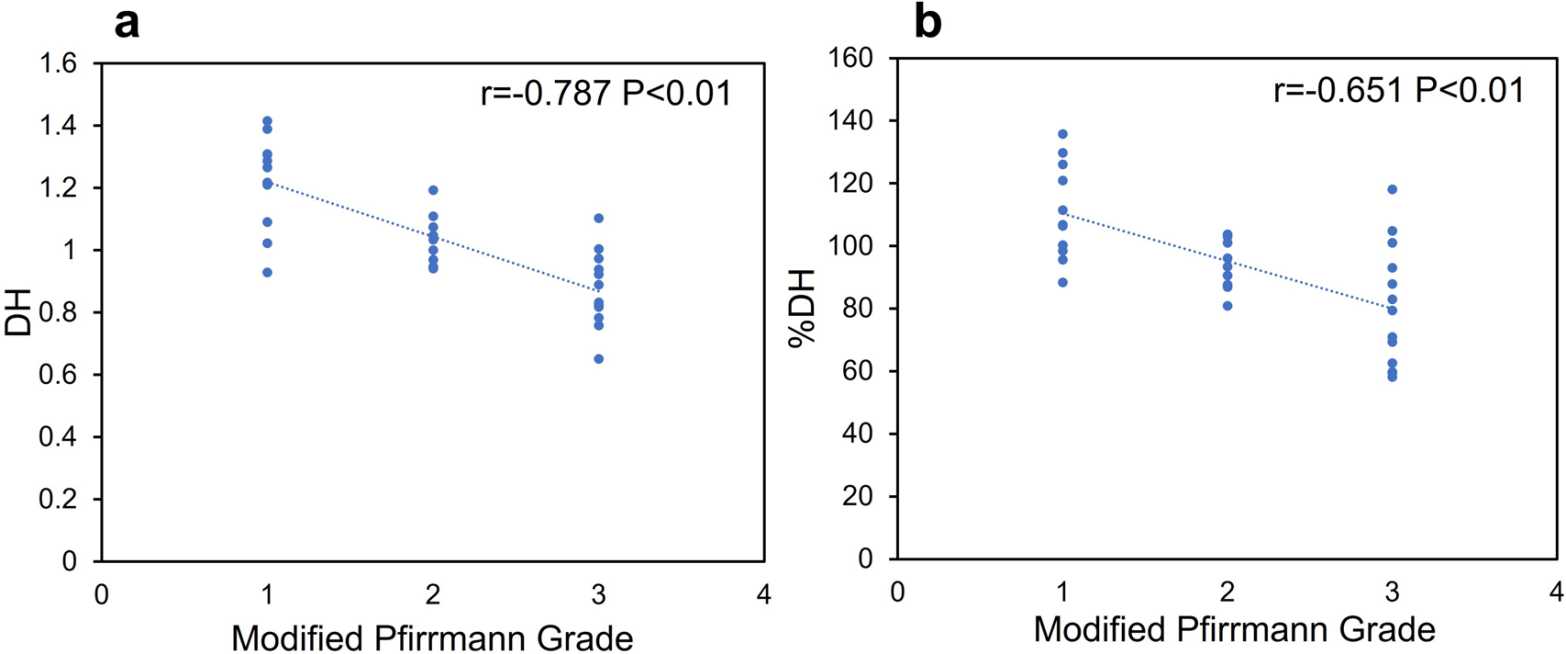
Correlation between MRI grading score and disc height (DH). MRI grading scores using the modified Pfirrmann grading system [20] were significantly correlated with **a** DH and **b** %DH. *r*, correlation coefficient

MRI grade scores were significantly correlated with DH (*r* =  − 0.787, *P* < 0.01) and %DH (*r* =  − 0.651, *P* < 0.01) on lumbar radiographs (Fig. 5).

### Histological assessment

**Fig. 6:**
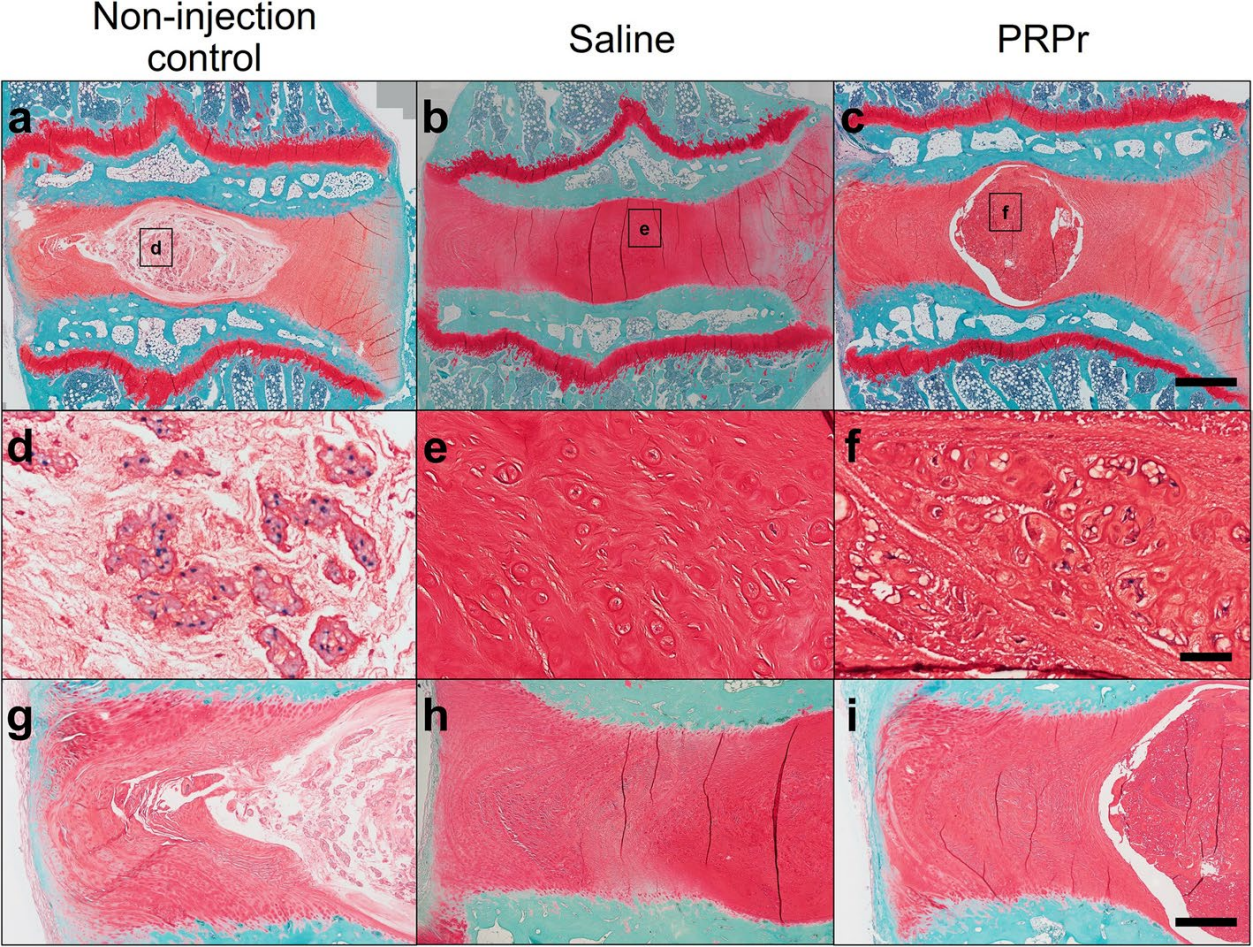
Typical histological changes of the non-injection control (**a**, **d**, **g**), saline (**b**, **e**, **h**), and platelet-rich plasma (PRP)-releasate groups (**c**,** f**, **i**) stained with Safranin-O. Moderate-to-severe degeneration was observed in the saline (**b**, **e**, **h**) groups. Regenerative changes were observed in the saline and PRPr groups, including cell cloning and rounded cells with intense pericellular matrix staining (**e**, **f**). However, the PRPr group displayed more viable cells and cell cloning than the saline group (**e**,** f**). **d**–**f** Magnification images of the nucleus pulposus area indicated by squares (**a**–**c**). **g**–**i** Magnification images of the annulus fibrosus area in **a**–**c**, respectively. Scale bars: 1 mm (**a**–**c**), 50 μm (**d**–**f**), 500 μm (**g**–**i**)

Representative histology (Safranin-O staining) of the non-injection control, saline, and PRPr groups at 16 weeks after condoliase injection is shown in Fig. 6. The non-injection control (L3-L4) discs showed the normal histological appearance of rabbit IVDs, displaying an intact AF with a regular pattern of fibrocartilage lamellae and a well-defined border between the AF and the NP. The NP consisted of numerous NP (vacuolated) cells within a fine cotton-like ECM (Fig. 6a, d, g). Intradiscal injection of condoliase induced significant changes in the histological appearance, especially in the NP. In the saline and PRPr groups, progressive degenerative changes were observed, including distortion of the NP shape, reduction of the NP area, condensation of the matrix, and loss of distinction between the AF and NP (Fig. 6b, c, e, f, h, i). No significant histological changes were observed in the endplates or subchondral bones among groups.

Regenerative changes were observed in both the saline and PRPr groups, including cell cloning and rounded cells with intense pericellular matrix staining (Fig. 6e, f). However, the PRPr group exhibited more viable cells and cell cloning than the saline group (Fig. 6f). The AF of the non-injection control disc, saline-injected discs, and PRP-injected discs showed a pattern of fibrocartilage lamellae (U-shaped posteriorly and slightly convex anteriorly), without ruptured fibers and a serpentine appearance in AF (Fig. 6g–i).

**Fig. 7:**
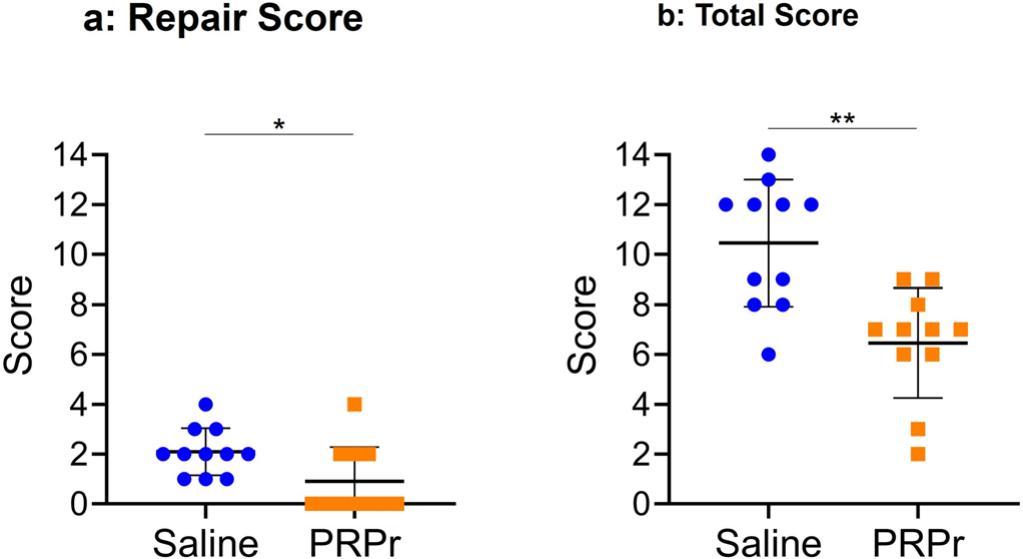
Histological grading scores. The histology was graded based on the standardized histopathological scoring system for rabbit IVD degeneration [21]. The repair score (**a**) is the sum of cell cloning and morphology. The total score (**b**) is the sum of the degeneration and repair scores. Data are presented as mean ± standard error of the mean (SEM). Non-injection control, *n* = 11 discs; saline, *n* = 11 discs; PRPr, *n* = 11 discs. **P < 0.01

The repair score of PRPr (0.9 ± 1.3) groups was significantly lower than that of the saline (2.1 ± 0.9) group (*P* < 0.05) (Fig. 7a). The total score of the PRPr group (6.5 ± 2.1) was significantly lower than that of the saline group (10.5 ± 2.4; *P* < 0.01) (Fig. 7b).

In the degeneration category, the NP area (*P* < 0.01), cell number (*P* < 0.05), and AF morphology (*P* < 0.01) scores were significantly lower in the PRPr group than in the saline group (Additional file 1). Endplate (EP) sclerosis or thickening was not identified in the two groups. In the repair score categories, the cell cloning score was significantly lower in the PRPr group than in the saline group (*P* < 0.01) (Additional file 1).

### Correlation between MRI grading score and histological score

**Fig. 8:**
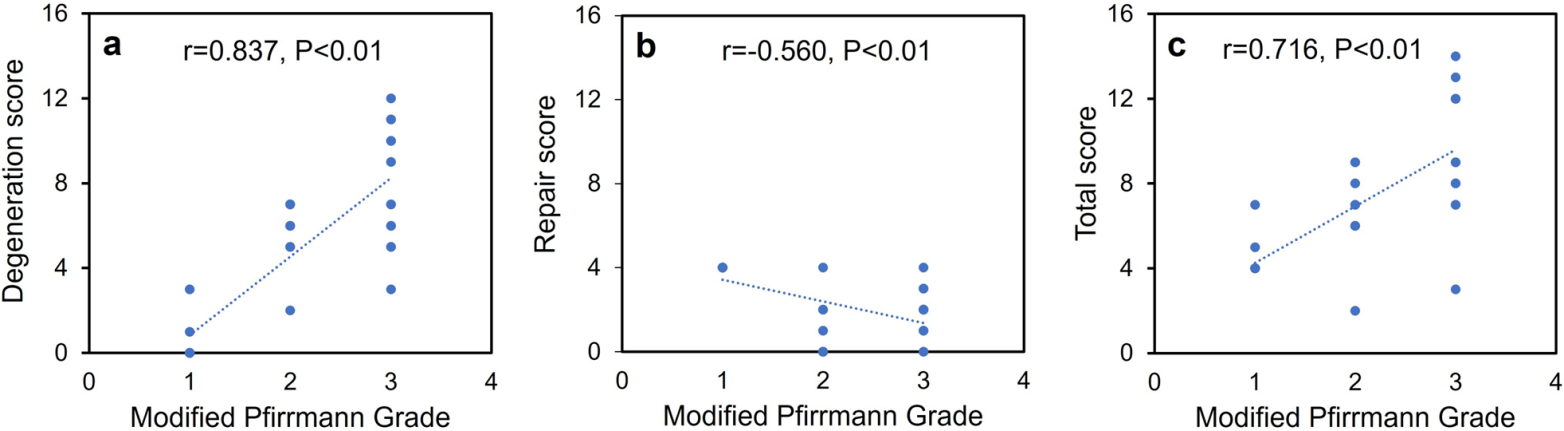
Correlation between MRI grading score and histological score. **a** Degeneration score. **b** Repair score. **c** Total score. *r*, correlation coefficient

The MRI grade scores were significantly correlated with the histological degeneration score (*r* = 0.837), repair score (*r* =  − 0.560), and total scores (*r* = 0.716) (Fig. 8).

## Discussion

This study evaluated the radiological and histological recovery of rabbit IVDs induced by intradiscal injection of PRPr after condoliase injection. Radiological, MRI, and histological assessments revealed that PRPr injection caused a significantly greater regenerative effect than saline injection on the degenerated disc induced by condoliase.

Yang et al. reported that condoliase injection at 0.015 U/disc gradually decreased rabbit DH during the follow-up, with a 40% decrease at 16 weeks [22]. Imai et al. showed that injecting condoliase at 0.01 U/disc into rabbit IVDs significantly decreased DH by approximately 34% at the 4-week time point and did not recover until 16 weeks [23]. The current study used a pharmaceutical-grade condoliase (Hernicore®, 0.0125 U/disc, the same dose clinically used for patients with LDH) for intradiscal injection into rabbit IVDs. DH was decreased to approximately 52% of baseline height at 4 weeks post-injection. However, contrary to earlier studies [22, 23], DH began to recover and reached approximately 76% at week 16, even in the saline-injected discs. Previous studies evaluated rabbit DH using the disc height index (DHI: ratio of disc height to vertebral height), and changes in the DH were expressed as %DHI that normalized to the baseline DHI [22, 23]. However, in the current study, the actual value of DH was directly measured by calibrating the radiological measurement using a 50-mm metal wire plated at the height of the spinal process of the rabbit lumbar spine, and the changes were expressed as %DH that normalized to the baseline. Differences in the measurement methods of disc height may have led to inconsistent results.

Several clinical studies on patients with LDH have shown spontaneous recovery of DH after injection of condoliase [9, 24, 25]. Banno et al. reported the 1-year outcomes of condoliase-induced chemonucleolysis for LDH in 60 patients. They showed significant DH recovery at 1 year following a substantial decrease in DH at 3 months post-injection [10]. Therefore, DH recovery in the rabbit animal model supports the spontaneous recovery observed during the clinical course of patients with LDH after the injection of condoliase (Hernicore®). Kobayashi et al. [26] reported that the age of LDH patients injected with condoliase of whom the remarkable restoration of DH was identified was significantly younger than those with poor DH restoration, suggesting that the regenerative potential of IVD against condoliase may be higher in the younger generation. The present study used 16-week-old rabbits corresponding to the late teens in humans, implying that young rabbit IVDs may have great regenerative potential to counteract condoliase injection.

Previous studies have reported the typical histological changes of IVD degeneration after the injection of condoliase, including matrix loss in the NP, reduction of cell number, and loss of distinction between the AF and NP border [22, 23]. In accordance with previous studies, the histology in the current study showed progressive degenerative changes, including distortion of NP shape, reduction of the NP area, condensation of the matrix, decrease in NP cell number, and loss of distinction between AF and NP in the saline groups at 16 weeks post-injection of condoliase. No histological changes were observed in the EP area, as previously reported [27].

Park et al. [28] also reported a decrease in chondrocyte-like cells in the NP with proteoglycan depletion seven days after the injection of condoliase, and electron microscopy revealed that collapsed cells (necrosis) were distributed in the NP. The results of the present and previous studies suggest that condoliase can enzymatically degrade the NP matrix, accompanied by cytotoxic effects that lead to degenerative changes in rabbit IVDs.

The present study showed that the intradiscal injection of PRPr at 4 weeks after condoliase injection significantly stimulated DH recovery (95.5% recovery to baseline value) compared to saline injection. In accordance with our results, Imai et al. [23] showed that intradiscal injection of recombinant human osteogenic protein 1 (rOP-1) into degenerated rabbit discs at 4 weeks after condoliase injection resulted in a significant increase in DH at 6 weeks after rhOP-1 injection; this change was sustained up to 16 weeks (> 90% recovery to baseline value). This suggests that the intradiscal administration of PRPr has an effect equivalent to that of rhOP-1 on DH restoration.

The extent of disc degeneration by MRI grading was milder in condoliase-induced degenerated discs injected with PRPr than in those injected with saline. Interestingly, the MRI grading score [20] showed a significant and strong correlation with the histological score of rabbit disc degeneration [21]. Histological analysis revealed that the NP area and cellularity scores were significantly better in the PRPr group than in the saline group. Furthermore, the cell cloning scores in the NP and inner AF were significantly higher in the PRPr-injected discs than in the saline-injected discs. Similar to the histology in the present study, Obata et al. [18] reported that the number of chondrocyte-like cells in the anterior AF and NP was significantly higher in discs injected with PRPr than in those injected with PBS in a rabbit annular puncture model [29]. The current histological results suggest that the intradiscal administration of PRPr may enhance cell proliferative activity and matrix remodeling within rabbit degenerated discs induced by condoliase, leading to the improvement in MRI grading and structural restoration of DH.

Two previous randomized controlled studies that determined the efficacy and safety of condoliase in patients with LDH showed that condoliase significantly improved neurological symptoms in patients with LDH [12, 13]. However, back pain, Modic type I changes, and a decrease in DH were frequently observed in the condoliase group. Recently, Banno et al. [9] reported the 2-year follow-up results of 67 patients with LDH who received condoliase. They reported that condoliase therapy was effective in 76.1% of the patients. However, 11.9% of the patients required additional surgery because of the ineffectiveness of this therapy. Progression of MRI-graded disc degeneration was observed in 57.1% of patients at three months; however, 30% recovered to baseline values at 2 years. These results suggest that condoliase therapy is effective in many patients with LDH; however, approximately 20% of patients may have worse clinical outcomes, poor DH recovery, or progression of MRI-graded disc degeneration.

The present preclinical study showed that the intradiscal injection of PRPr significantly enhanced DH recovery and MRI-graded disc degeneration. Hence, PRPr injection therapy may be indicated for patients with poor recovery from disc degeneration or worse clinical outcomes after condoliase treatment. PRP and PRPr have been reported to have significant anti-inflammatory effects on IVD cells in vitro [14]. A previous clinical trial reported that the intradiscal injection of PRPr relieved LBP and improved disability and quality of life during 60 weeks of observation in patients with discogenic LBP [17], suggesting that PRPr injection may also improve the clinical symptoms of patients with LDH in whom condoliase was ineffective.

Previous histological and biochemical studies showed that the enzymatic activity of condoliase lasts up to 4 weeks after the intradiscal injection in rabbit IVDs [24]. Therefore, PRPr was intradiscally administrated 4 weeks after the injection of condoliase in the current study. In the clinical setting, PRPr should be injected into the degenerated discs 4 weeks after the condoliase treatment in patients with lumbar disc herniation.

The current histological study showed that intradiscal injection of PRPr following condoliase treatment did not induce disc herniation in the rabbit IVDs. The previous randomized clinical trial showed that intradiscal injection of PRPr was safe and maintained significant improvement in pain, disability, and quality of life without disc herniation or worsening disc bulging during 60 weeks of follow-up in patients with discogenic LBP [17, 30]. Therefore, the authors speculated that the intradiscal injection of PRPr following condoliase treatment in patients with lumbar disc herniation is also safe and does not lead to the recurrence of disc herniation. Nevertheless, clinicians should consider the possibility of hernia recurrence following PRPr injection.

This study has several limitations. First, this study compared the saline and PRPr groups; however, intradiscal injection of saline 4 weeks post-injection of condoliase may have some effects on disc cells. Therefore, only condoliase-injected discs should be assessed to evaluate the natural history of condoliase injection. Second, the observation period of this study was 16 weeks after the injection of condoliase. A long-term follow-up study is needed to evaluate the long-term results of condoliase and subsequent PRPr injections in future research. Third, immunohistochemical analysis of the expression of anabolic, catabolic, and matrix molecules would help further understand the biological effects of PRPr in degenerated rabbit IVDs, which should be evaluated in future studies. Lastly, potential biomechanical and anatomic differences exist between the rabbit (quadrupedal animal) and human (bipedal animal) lumbar spines. Sixteen-week-old New Zealand white rabbits used in this study are equivalent to a 10- to 12-year-old human. Hence, the regenerative capacity of young rabbit IVDs used in this study might be higher than that of human IVDs of 30–50 years of age, at which lumbar disc herniation usually occurs. Therefore, the regenerative effects of PRPr identified in the rabbit IVDs may not directly reflect the responses of PRPr in human degenerated discs.

## Conclusions

Intradiscal injection of PRPr induced significantly greater DH recovery than saline injection in rabbit IVDs degenerated by condoliase injection. MRI and histological analyses revealed milder degenerative changes in the PRPr group than in the saline group. These results suggest that PRPr has an excellent potential to enhance the regeneration of degenerated rabbit IVDs induced by condoliase. The results of this preclinical study suggest that PRPr injection therapy may be indicated for patients with LDH who have poor recovery from disc degeneration after chemonucleolysis treatment with condoliase.

### Supplementary Information

**Additional file 1.** Histological grading scores of nine categories. The histology was graded based on the standardized histopathological scoring system for rabbit IVD degeneration [21]. The degeneration score was calculated using the sum of seven categories: NP shape, NP area, matrix condensation, cell number, border appearance, AF orphology, and endplate (EP). The repair score is the sum of cell cloning and morphology. Data are presented as mean ± standard error of the mean (SEM). Non-injection control, *n* = 11 discs; saline, *n* = 11 discs; PRPr, *n* = 11 discs. *P<0.05, *P<0.01.

## Data Availability

The datasets used and/or analyzed during the current study are available from the corresponding author upon reasonable request.

## Abbreviations

AF: Annulus fibrosus
ECM: Extracellular matrix
IVD: Intervertebral disc
LBP: low back pain
LDH: lumbar disc herniation
MRI: Magnetic resonance imaging
NP: Nucleus pulposus
PRP: Platelet-rich plasma
PRPr: Platelet-rich plasma releasate
