# Supplementary figures and images for: Regenerative effects of platelet-rich plasma releasate injection in rabbit discs degenerated by intradiscal injection of condoliase
Source: Arthritis Res Ther. 2023 Nov 8;25:216. doi: 10.1186/s13075-023-03200-w (PMC10631205; doi:10.1186/s13075-023-03200-w)

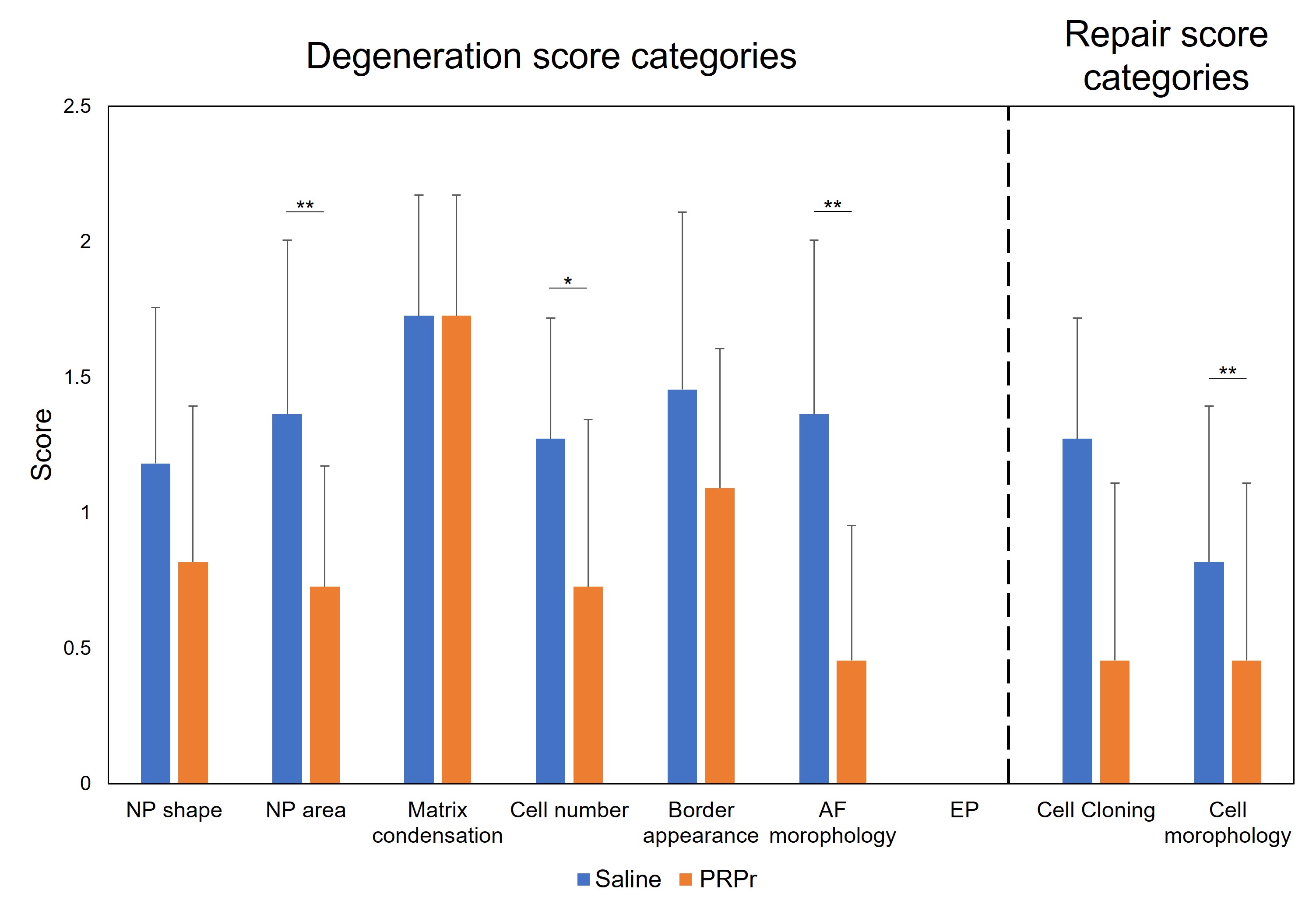

Supplement: Supplementary file 1 — Additional file 1. Histological grading scores of nine categories. The histology was graded based on the standardized histopathological scoring system for rabbit IVD degeneration [21]. The degeneration score was calculated using the sum of seven categories: NP shape, NP area, matrix condensation, cell number, border appearance, AF orphology, and endplate (EP). The repair score is the sum of cell cloning and morphology. Data are presented as mean ± standard error of the mean (SEM). Non-injection control, n = 11 discs; saline, n = 11 discs; PRPr, n = 11 discs. *P<0.05, *P<0.01. [file 13075_2023_3200_MOESM1_ESM.jpg]
